# DNA methylation of RAMP1 gene in migraine: an exploratory analysis

**DOI:** 10.1186/s10194-015-0576-7

**Published:** 2015-10-26

**Authors:** Dongjun Wan, Lei Hou, Xiaofei Zhang, Xun Han, Min Chen, Wenjing Tang, Ruozhuo Liu, Zhao Dong, Shengyuan Yu

**Affiliations:** Department of Neurology, Chinese PLA General Hospital, 28 Fuxing Road, Haidan District, Beijing, 100853 China

**Keywords:** Migraine, DNA methylation, RAMP1

## Abstract

**Background:**

Receptor activity modifying protein 1(RAMP1) is a key receptor subunit of calcitonin gene related peptide (CGRP) playing a critical role in migraine. But variations in RAMP1 gene have not been found to link with migraine. Still it is elusive that DNA methylation at RAMP1 promoter is associated with migraine.

**Methods:**

A total of 51 blood DNA samples from 26 patients with migraine and 25 matched healthy controls were collected, extracted and treated with bisulfate. Subsequently DNA methylation levels at RAMP1 promoter region were measured using Sequenom Mass ARRAY systems.

**Results:**

Among 13 detected CpG sites or units at RAMP1 promoter region, there were no significant differences between the migraine and control groups, but indicating a low methylation trend overall in migraine group (total average methylation level: 8.41 % ±1.92 % vs. 9.90 % ± 3.88 %, *p* = 0.197). Stratification analysis showed that methylation level at (+25, +27, +31, related to the transcription start site) CpG unit was higher in migraineurs with migraine family history compared to those without (13.92 % ± 5.97 % vs. 8.77 % ± 6.61 %, *p* = 0.034), and methylation level at (+89, +94, +96) CpG unit was lower in migraine female than that in healthy female (2.18 % ± 1.91 % vs. 5.85 % ± 5.41 %, *p* = 0.02). For female with methylation level at (+89, +94, +96) CpG unit below 3.50 %, the probability of being a migraine patient was significantly higher than those with methylation level above the threshold (OR: 7.313; 95%CI: 1.439-37.164).

**Conclusions:**

This study provides the first evidence that DNA methylation at RAMP1 promoter might play a role in migraine. A low methylation trend overall was presented in migraine subjects, and two CpG units were observed to link with positive migraine family history and female migraine, respectively. Lower methlytion level at (+89, +94, +96) CpG unit may be a risk of migraine in females.

## Background

Migraine is a common primary headache disorder ranked as the 7^th^ disabling condition by WHO [1], which affects approximately 9.3 % population in China [2]. Although more current advances in migraine have been obtained, the complex mechanism of migraine is far from fully understood and it is still lacking biomarkers available for prediction of migraine susceptibility, differential diagnosis and the prediction of response to treatment [3].

Epigenetic processes are thought to contribute to the etiology of many diseases including cancer and vascular disease, which are mediated through interfering potential transcription and gene expression [4, 5]. DNA methylation, a basic epigenetic modulation, mainly occurs at cytosine residues in the CpG dinucleotides within promoter region, resulting in chromatin compaction and gene silencing [6]. Also epigenetic alterations have provided a potential cancer marker, which could be used for the early examination as well as prediction of therapeutic responses [7]. Numerous studies from animal experiment suggest that DNA methylation accounts for chronic pain [8]. Epigenetic modulations are supposed to participate in etiology of migraine, but few clinical evidence suggest that DNA methylation modulation is involved in migraine [9].

Receptor activity modifying protein 1 (RAMP1) is a key receptor subunit of calcitonin gene related peptide (CGRP), which functions as an important neural transmitter implicated in migraine [10]. Emerging evidence indicated that changes in the expression of RAMP1 can affect the sensitivity of cell to CGRP [11]. The Nestin/RAMP1 transgenic mice, over-expressed human RAMP1 in central neurological system (CNS), can mimic some features of migraine, such as photophobia and allodynia, when intracerebroentricular administration of CGRP [12, 13]. These suggested RAMP1 would have a potential role in migraine. However, genetic polymorphism studies and genome-wide associated studies failed to link migraine with variations in RAMP1 gene [14, 15]. Although at the promoter region of RAMP1, these sequences contain a number of CpG dinucleotides, few attempts are to investigate the relationship between DNA methylation of RAMP1 gene and migraine.

In this study, our aim is to explore whether methylation pattern at the promoter region of RAMP1 gene in peripheral leukocyte is associated with migraine using quantitative methylation approach.

## Methods

### Subjects

Patients with migraine were recruited at International Headache Center of Chinese PLA General Hospital from June to November in 2014. Matched healthy controls were enrolled from hospital staff and non-consanguineous relatives of patients. All participants had signed informed consents according to the Ethics Committee of PLA General Hospital.

Patients were inquired medical history and underwent a detailed physical and neurological examination, as well as screening head magnetic resonance imaging or computed tomography. A detailed information of each participants were recorded including demographic characteristic, headache features [pain severity (visual analog scale, VAS), duration of headache and accompanied symptoms during attacks], family history and medication for treatment. Inclusion criteria for migraineurs were: (i)definite migraine with aura or without aura were diagnosed according with International Classification of Headache Disorders, 3rd edition (beta version) [16]; (ii)no systematic disease and not using preventative medicine; and (iii)exclude pregnant or breast-feeding women. Healthy controls had no headache history and systematic disease.

### Blood sample collection and DNA isolation

For all participants, EDTA blood samples were drawn from the cubital vein and stored at −80 °C until analysis. Blood DNA were extracted by standard protocols, quantified by NanoDrop 2000(Thermo Scientific, USA). Each DNA sample was aliquoted and stored at −20 °C for further methylation analysis.

### DNA methylation analysis with sequenom mass ARRAY systems

CpG islands of the RAMP1 gene (NC_000002.12) were analyzed in a 3000 bp region, which comprised putative promoter sequences and the first exon (−2000 bp to 1000 bp, related to the transcription start site, TSS) using CpG Island Searcher (http://cpgislands.USC.edu). The primer was designed by Sequenom EpiDesigner (Sequenom, San Diego, CA). Production of 505 bp contains the region (−300 to 205, related to TSS).

Quantitative methylation was analyzed in BGI TECH SOLUTIONS (LIUHE BEIJING) Co. Ltd in Beijing, China. 500 ng DNA sample was treated with bisulfate using the EZ DNA Methylation Gold kit (ZYMO RESEARCH CORP., Irvine, CA, USA) according to the manufacturer’s instructions. After bisulfate conversion, PCR amplification of the CpG islands region was performed: 94 °C, 4 min; 45 cycles of 94 °C, 20 s, 56 °C,30 s, 72 °C, 1 min; then 72 °C,3 min. For the interested CpG islands, primers were: forward primer 5’-GGGATTTTTGGATTTATTTATTTAGG-3’, reverse primer 5’-TTAACCTTTTACAAAACAAACACCA-3’. A T7 promoter tag (CAGTAATACGACTCACTATAGGGAGAAGGCT) was added to the reverse primer. Followed treatment with shrimp alkaline phosphatase, T7 polymerase/ribonuclease A and desalting, PCR production was conducted with Mass ARRAY EpiTYPER system (Sequenom, CA, USA) according to manufacturer’s instructions. Fully methylated DNA and water were as a positive and negative control, respectively. Percentage of methylation of each CpG unit or single CpG site was calculated using EpiTYPER software version 1.0.

### Statistical analysis

Normality distribution of variables was examined using Kolmogorov-Smirnov test. The group differences of age and gender were compared by independent *T* test and exact *x*^2^ test, respectively. The non-parametric Mann–Whitney test was performed to evaluated group differences with regard to DNA methylation level. Since the CpG sites within RAMP1 gene promoter region are not independent but correlated with each other [17], and the sample size was small, we did not perform the strict Bonferroni correction or other conservative approach. The receiver operating characteristic (ROC) curve and Youden criterion were used to determine the optimal cut-off point of methylation level. Odds ratio (OR) and 95 % confidence interval (CI) were computed to estimate the risk factor. In addition, correlations between average methylation level and age, VAS scores, as well as migraine duration were examined by Pearson test. *P* < 0.05 was set as statistical significance.

All analyses were conducted with SPSS for Windows (version 20.0, Chicago, IL, USA).

## Results

### Subjects demographics

A total of 26 patients with migraine (male: 9, female: 17) and 25 healthy volunteers (male: 11, female: 14), ranging from 14 to 58 years old, were recruited. Of 26 migraineurs, 13 had family history of headache and 13 were accompanied photophobia during attacks. The characteristics of participants are presented in Table 1. There were no significant differences in gender and age between migraine and control groups.

**Table 1:** Demographics of subjects with migraine and healthy controls

	Migraine	Control
n.	26	25
Age(mean ± SD,years)	35.0 ± 6.9	31.8 ± 7.0
Gender M/F	9/17	11/14
With aura	5	
With migraine family history	13	
With photophia	13	
Duration of migraine (mean ± SD, years)	12.69 ± 8.85	
VAS score (mean ± SD)	7.08 ± 1.82	

*F* female; *M* male, *VAS* visual analog scale

### Methylation patterns

Overall, there was no significant difference in the DNA methylation level among 13 detected CpG sites or units at the RAMP1 promoter region between migraine and control groups. Nevertheless, the results only indicated a low methylation trend in migraine versus control group (total average methylation level: 8.41 % ±1.92 % vs. 9.90 % ± 3.88 %, *p* = 0.197).

**Fig. 1:**
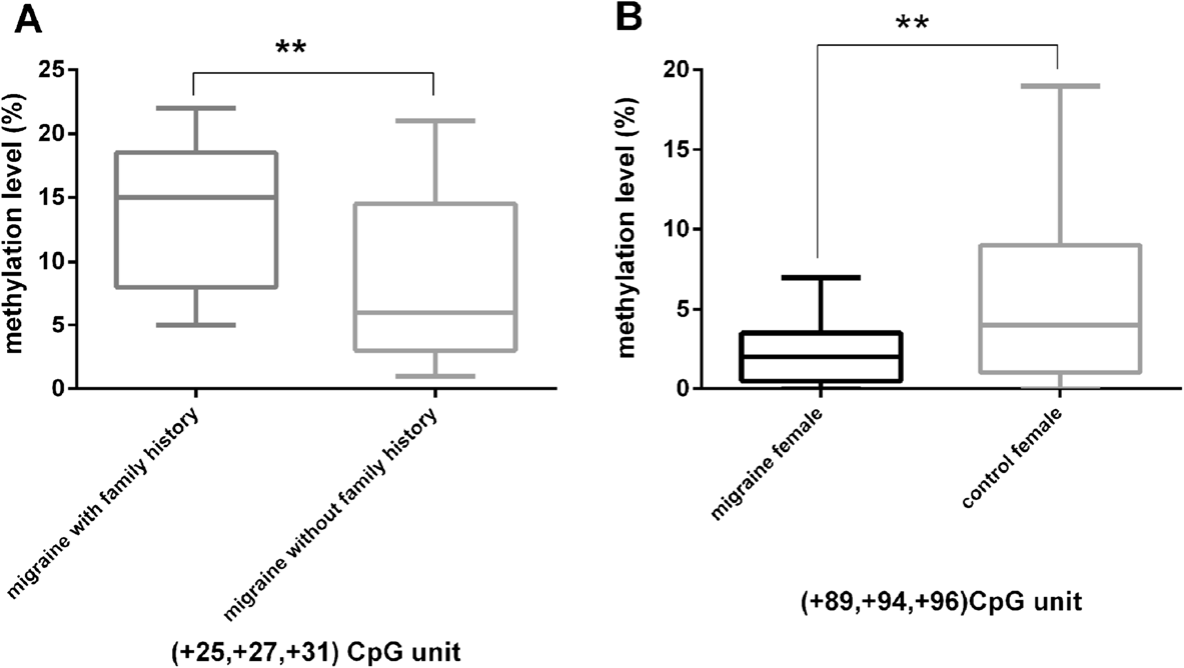
**a**: Methyaltion level at (+25, +27, +31, related to TSS) CpG unit is higher in patients with migraine family history compared to those without migraine family history. **b**: Methylation level at (+89, +94, +96) CpG unit is lower in migraine female than that in healthy female. The plots show methylation values of CpG unit at RAMP1 promoter region. There are significant differences in methylation level between the two groups (**p < 0.05)

Further stratification analysis showed that methylation level at (+25, +27, +31, related to TSS) CpG unit was higher in migrianeurs with migraine family history than those without migraine family history (13.92 % ± 5.97 % vs. 8.77 % ± 6.61 %, *p* = 0.034) (Fig. 1a).

Interestingly, we found that methylation level at (+89, +94, +96) CpG unit was significantly lower in migraine female patients than that in healthy female volunteers (2.18 % ± 1.91 % vs. 5.85 % ± 5.41 %, *p* = 0.02) (Fig. 1b), but not among male participants. To evaluate the migraine risk of female, 3.50 % methylation level at (+89, +94, +96) CpG unit as an optimal threshold was achieved according to ROC and Youden index, displaying sensitivity 0.765 and specificity 0.692. For female with methylation level below 3.50 %, the probability of being a migraine patient was significantly higher than those with methylation level above the threshold (OR: 7.313; 95%CI: 1.439-37.164).

Stratification analysis by photophobia was performed among migraineurs. Differences in methylation pattern were not observed between photophobia and non-photophobia group. Furthermore, no significant correlation was found between age within all participants and average methylation level. Yet, VAS score and duration of migraineurs were not related with average methylation level.

## Discussion

RAMP1 is a single-transmembrane receptor subunit, conferring ligand specificity, necessary for the function of CGRP [18, 19]. Clinical observations reveal that injection of CGRP could introduce a delayed migraine-like headache in migraineurs, but not in migraine-free controls [20], which indicates the regulation of RAMP1 gene could been implicated in migraine susceptibility. Several transgenic and expression studies also demonstrate that RAMP1 is involved in migraine [12, 21, 22]. However, previous genetic variant and genome-wide associated studies did not show any positive correlation between RAMP1 and migraine [14, 15]. The underlying mechanism is still unkown. Epigenetic modifications have been proved to play a crucial role in complex chronic disorders [23, 24]. We hypothesized that epigenetic modulation could participate in the pathological mechanism of migraine, and expected that differential DNA methylation of RAMP1 in leukocytes could provide potential epigenetic biomarkers for the identification and diagnosis of migraine.

However, our results showed that no significant difference of methylation of each examined CpG unit or site within the region (−300 to 205) was observed between patients with migraine and healthy controls except for a low average methylation trend in migraine group. Furthermore, due to animal experiments revealing a link between overexpression of hu-RAMP1 and photophobia [13], we are sought to discover the differences of methylation pattern. Unfortunately, we found no statistical difference between photophobia and non-photophobia subgroups. Because it is, to our knowledge, the first time to investigate the methyaltion pattern of RAMP1 in migraine, we could not perform an accurate power calculation. Taking account of the small size of samples, future studies with a larger cohort to increase the power to obtain significant correlation are required.

Most importantly, we found that (+25, +27, +31) CpG unit and (+89, +94, +96) CpG unit were associated with positive migraine family history and female migraine, respectively. When methlytion level of (+89, +94, +96) CpG unit decreased, the risk of migraine in females seemed increased, but not in male. Therefore, it is being thought whether this could be regarded as an epigenetic biomarker to predict female migraine risk, analogous to tag SNPs, needs enlarged-size samples to be warranted. Being lack of the exact mechanism of DNA methylation implicated in migraine, from the present data, it is difficult to interpret the differences of DNA methylation at RAMP1 gene promoter within migraine subgroups. Sex-specific difference in mRNA levels of RAMP1 have been observed in rat migraine model [25], as also have been found in the light-aversion behavior in transgenic hu-RAMP1 mice [26]. Moreover, recent methylome study also reveals more genes with sex differences in DNA methylation [27]. Whether female predominance of migraine is associated with sex-biased methylation, remains to be elucidated.

Finally, we acknowledged that methylation pattern in peripheral leukocyte could not really represent the similar trend among the tissues of CNS, owing to tissue-specificity in DNA methylation [17]. Nevertheless, several studies have suggested that peripherally epigenetic changes could be used as markers for research purpose in many CNS disorders [28–30]. Zill P and his colleagues analyzed the DNA methylation pattern of ACE gene in peripheral leukocytes and found that aberration of DNA methylation at ACE promoter may be an underlying cause of major depression [29]. In addition, methylation status of repetitive elements in blood of Alzheimer’s disease may be novel markers for risk stratification [28]. These studies further support that disease-specific DNA methylation changes may occur in peripheral blood and provide a specific profile of disease.

There were several limitations to be considered. First, based on the limited samples of the selected population in our present study, our findings and subsequent conclusions need to be further warranted. Second, owing to undetecting the expression of RAMP1 in the leukocytes, it is difficult to assess whether and what extent of methylation level affect the expression of RAMP1. Third, because of obvious difficulties in obtaining the specific tissue derived from trigeminovascular or cerebral biopsies, we cannot provide direct evidence that epigenetic modulation is involved in migraine. Finally, peripheral blood DNA is derived from multiple cell lineages. Therefore the differences in numbers of subpopulation might result in distinct DNA methylation patterns.

## Conclusions

This study provides the first evidence that DNA methylation at RAMP1 promoter might play a role in migraine. Even though we found no significant differences of DNA methylation pattern between migraine patients and healthy controls, a low methylation trend overall was presented in migraine group, and two CpG units were observed to link with positive migraine family history and female migraine, respectively. The methylated marker might be helpful to predict the migraine risk. Still, the results need larger samples to be further validated and the molecular mechanism underlying remains to be investigated.
